# The Principles of Surgery

**Published:** 1845-01

**Authors:** 


THE PRINCIPLES OF SURGERY. By *James Miller*, F.R.S.E. F.R.C.S.E. Professor of Surgery in the University of Edinburgh, Surgeon to the Royal Infirmary, &c. &c.

THIS work "contains the substance of the Author's systematic Lectures on this subject," and is "intended to exhibit a condensed view of the Principles of the Healing Art." Mr. Miller appears to have written it chiefly as a text-book, as well as of reference for his own pupils, yet hopes it may prove of service to others, "as a concise exposition of the Science of Modern Surgery."

Our brethren of the North appear to feel themselves called upon to shew the professional world that the position of honor, to which they may recently have been elevated, has not been unworthily bestowed: thus, among others, we have the two Surgeons from Scotland, now holding high office as Teachers and Hospital Surgeons in the Great Metropolis, shining forth as authors of works on Surgery; and, within a year or two afterwards, the subject of our present remarks appears. We strongly object to the too-prevailing practice of professional works being written solely for the sake of the author, the only justifiable reason—we had almost written excuse—for a medical practitioner to obtrude his work upon the public, is the conviction of its usefulness, and that such a volume is really a desideratum: this can hardly be conceded to both the works to which allusion has been made; and the mere mention of a third treatise on surgery from the same school, and within so short a period, certainly gave rise to some feeling allied to regret. This feeling was however completely removed by a reference to the book itself; for, while we found little more than detail, or practice as it is generally termed, in Mr. Liston's and Mr. Fergusson's works, we here meet with general principles, with more expanded views of the real science of disease, doctrines upon which the details of practice may easily be engrafted; hence, although it probably will not be studied by the pupil preparing for his examination so readily as more practical essays, it will be received by the more advanced student and the practitioner as an important and valuable addition to his library, condensing, as it does, what is really known of pathology up to the present time.

Although we can speak very favourably of Mr. Miller's production, and strongly recommend it, not only to the perusal, but to the careful study, of all medical men, we shall take the liberty, in the review of the book, to apply our strictures, we hope in all fairness, where we think them due.

A "Historical Notice," that is a History of the Progress of the Art of Surgery, which was written by Mr. Miller for the Encyclopædia Britannica, introduces the work. We have no objection to an occasional dissertation upon the origin and advancement of our art appended to volumes, though professedly written upon its science or practice, inasmuch as we fear the medical student and practitioner are in general both sadly deficient in the literature of our profession, and not nearly sufficiently acquainted with the writings, or even the names, of the worthies that have preceded us; we should however lament to have such an introduction generally imposed upon medical works as unnecessary and irrelevant. This sketch of the history of surgery is very well composed, and Mr. Miller has given honor where honor is due, and has restored the credit to some of the older surgeons, which it has been the unworthy endeavour of a few moderns to appropriate to themselves. A spirit of truthfulness and candour pervades this history, which is very refreshing. It was hoped that all attempts to maintain the unnatural and arbitrary divisions of medicine and surgery—at least so far as the *study* of the profession was implicated, whatever might be thought with regard to its *practice*,—were at an end, never to be revived, but the late recently chartered, and proposed legislative enactments, appear to have the tendency to perpetuate this baneful anomaly; with these impressions we read with gratification that "it's (surgery's) complete separation from medicine would now be attended with the utmost difficulty; (would it not be impossible?) nor is it desirable that the attempt should be made, because its success, however partial and imperfect, would be most hurtful to both. They are now, and it is to be hoped ever will remain, one and inseparable. Their principles are the same throughout, and the exercise of their different branches requires the same fundamental knowledge; but their details are so numerous and intricate as to render it most difficult, if not impossible, for any one individual to cultivate all with equal success." "The separation, however, is not one of acquirements, but merely of practice. It should never be forgotten, that the physician, before he can be either accomplished or successful in his profession, must be intimately conversant with the principles, if not with the practice, of surgery. And most certainly no one can ever lay just claim even to the the title of surgeon, far less hope for eminence or success, unless he be equally qualified to assume both the appellation and employment of the physician." We pass over the consideration of the historical notice the more readily, as Mr. Adams's translation from the Greek of the "Seven Books of Paulus Ægineta, with a commentary embracing a complete view of the knowledge possessed by the Greeks, Romans and Arabians, on all subjects connected with Medicine and Surgery," from which Mr. Miller has drawn some of his particulars, is, or soon will be, in the possession of most of the profession, it being now in the course of publication by the Sydenham Society.

Our author has divided his work into four Sections without intending, it is presumed, to convey a nosological arrangement. The first Section includes what he has termed "Elementary Disease;" this is subdivided into six Chapters, including "Perverted Action of the Bloodvessels," Inflammation and Congestion; "Perverted Action of the Nerves," Irritation, &c.; "Perverted Action of the Absorbents" without inflammation; "Suppuration;" "Ulceration," including Granulation & Cicatrization; and "Mortification." The second Section treats of "Perverted vascular action in certain tissues;" first in the Integument; second, in Bone; third, Diseases of the Joints; fourth, Diseases of the Arteries; fifth, of the Veins; sixth, Hæmorrhage; seventh, Affections of the Lymphatics; eighth, of the Nerves. The third Section contains "Perverted Nutrition," which has one Chapter on Tumors, Benign, Malignant, Encysted, and of different tissues. The fourth Section includes "Injuries;" first, Wounds; second, Burns and Scalds; third, the Effects of Cold; fourth, Fractures; fifth, Dislocations; sixth, Sprain and Rupture of Muscle and Tendon; seventh, Bruise.

Without saying that Mr. Miller has adopted the best arrangement possible of the principles of disease, we think the above is convenient to facilitate the student's study, beyond which we feel that the hitherto attempts at nosology are little more than useless, as, in the present deficiency of knowledge of the true essence of disease, such dove-tailing of one affection upon another cannot be made upon scientific principles. We cannot however recognise the improvement of interposing irritation, and simple absorption, between inflammation and its products; surely it is better to treat of diseases and changes in structure, which are known to depend upon each other, as nearly as can be consecutively.

The Chapters on Inflammation and its consequences are the most important, from the perusal of which we have derived considerable pleasure and instruction. Mr. Miller defines inflammation as "a perverted condition of the blood and blood-vessels of a part interrupting its healthful function, and changing its normal structure; ordinarily attended with redness, pain, heat, and swelling; and inducing more or less disturbance of the general system." It is difficult to give an unexceptional definition of any disease, and of inflammation in particular; probably that by Galen, and which the above includes, is yet the most simple and comprehensive. Our author objects to the term inflammation being made to include simple increased action, which may, and often does, readily subside upon the removal of the cause, as well as the destructive disease which leads to suppuration, ulceration, and mortification; the "one," he says, "is something not at variance with health, the other is inflammation." Yet he states it is but "a transition gradually effected;" the condition of the blood-vessels is the same in the "blush of shame, or the red spot of hectic" as in the commencement of that inflammation which is to terminate in the destruction of the part, however much they may differ in the sequel. We prefer Mr. Miller's subdivision of the "transition into the three stages;—1. Simple Vascular Excitement; 2. Active Congestion; 3. True Inflammation," to his objection, that they should be "declared the offspring of one common parent inflammation." In simple vascular excitement "the natural function of the part is exalted; if this be secretion, the secreted fluid is increased in quantity, yet with its normal characters scarcely, if at all, changed; nutrition is exalted; and the fibro-cellular tissue is fuller than before, giving slight increase of bulk." "This is not inconsistent with health, but rather its mere exaltation—synonymous with the *Vital Turgescence* of some physiologists. The part contains an increased amount of blood; its circulation is unusually active, and there is a marked tendency to increased exudation, partly serous, partly of a plastic kind." This very commencement of inflammation often exists in a more simple degree than described in the above quotation, and entirely subsides, or proceeds to disorganizing disease.

In " Active Congestion the vascular connection extends on the cardiac side of the affected part; the arterial trunks feeding it have partaken in the general excitement, are begun to enlarge, and are pulsating with an unwonted energy. More and more blood is sent down to the part, and the capillaries and minute arteries begin to give way beneath their burden; hitherto they were simply dilated, retaining their tone, and controlling the circulation of their contents; but now enlargement is about to be merged in overdistension, the vascular coats gradually parting with their tone." " The circulation loses its acquired rapidity, and becomes slower even than in health." Then is described the change which takes place in the blood, and Mr. Miller concludes—" Thus is constituted *Active Congestion;* the arterial trunks in increased play; the amount of blood in the part still farther augmented; its vessels beginning to be overdistended, and losing tone thereby; its circulation becoming slow; its blood undergoing change, the fibrin especially being increased, both in quantity and plasticity; function and nutrition perverted. We are leaving the confines of health, and have, indeed, already made some progress into the territory of disease."

" In *True Inflammation* the languor of circulation approaches stagnation, and at some points this has actually occurred; every part of the distended capillaries is occupied by crowded coloured and colorless corpuscles; partly, it may be, from increased attraction between the former and surrounding parenchyma, partly by accumulation and adhesion of the latter to each other and to the capillary walls. The altered liquor sanguinis is exuded in profusion. The capillaries also give way in their coats, and from the lesion blood is extravasated in mass. Suppuration is in progress by extra-vascular degeneration of the fibrinous effusion, or else by a secretive elaboration of it, ere yet it has left the vessel. Breaking up and disintegration of texture ensue, according to the extent of extravasation and suppuration; and the disintegrated texture is commingled with the effusion. The formative power has ceased, and the opposite condition, a tendency to disintegration, from diminution of vitality, has become established. Disorder of function is complete; secretion, for example, being in the first place arrested, and, when restored, more vitiated than before."

" The inflammatory change of the blood is important. 1. The liquor sanguinis is increased in relative quantity, and its serum is said to contain an unusual amount of albumen. 2. The fibrin is increased in quantity, both actually and relatively, to the red corpuscles; the vital attraction between its component particles, tending to aggregation, is also augmented. During active congestion its plasticity was increased, but now it becomes more and more aplastic. The proportion of serum is diminished, probably in consequence of effusion. 3. The red corpuscles are relatively diminished in number; and their tendency to aggregation is augmented. 4. The colourless or 'lymph globules' are greatly more numerous, but whether by new formation, or by mere accumulation in the part, has not yet been determined. They incline, not only to aggregation, but also to adhere to the sides of the vessels; thus increasing, or according to some, causing the tendency to stagnation of the blood." "This alteration of the blood, begun in the second, and completed in the third, or true inflammatory stage, is at first a local act, effected in the part inflamed; but this laboratory, if continued thus in operation, ultimately involves the whole circulating fluid in similar change."

These extracts will shew the manner in which the Theory of Inflammation is treated; to illustrate the fact of the disease extending from the centre, Mr. Miller has furnished a diagram of three circles one within the other, the internal denoting Inflammation, the second Active Congestion, and the external, Simple Vascular Excitement. In reference to the disputed question "whether inflammation is the result of an increase or diminution of vital strength in the part," the author states the fact "to lie midway between the disputants;" the action to commence with excitement, probably of short duration, to be succeeded by growing debility and much ultimate prostration. From which "a part once truly inflamed, never altogether recovers, but ever remains both more prone to action, and less able to control it; a fact which it is of much importance that both patient and practitioner should bear in remembrance."

While discussing the local symptoms, that of swelling is illustrated by a diagram similar to the one already alluded to, showing at a view the situation of the different effusions. Mr. Miller has not mentioned Hunter's experiment, nor his conclusion from it, that the heat of the inflamed part is not really raised above the natural standard, that it is only apparent; but describes this important condition as an established fact; we believe that John Hunter's experiment cannot be depended upon, nor consequently his conclusion, and that the heat, both apparent and real, is augmented in inflammation. Besides the four cardinal local signs, as originally laid down by Galen—Redness, Swelling, Heat, and Pain, the author has added Throbbing, Increased Sensibility, and Disorder of Function; the two first-named symptoms may be properly ranged under the head of Pain, being causes of that prominent sign, yet useful in the catalogue as denoting peculiarities of the pain and their causes; the throbbing arising from the mechanical impediment to the circulation of the blood increasing the pulsation of the arteries of, and leading to, the part; and the increased sensibility denoting the condition of the nerves. "Obviously this (latter symptom) is a wise and beneficial arrangement," "not only to suggest the propriety of rest, but also to compel its adoption. How lamentably destructive might not inflammation prove were it unaccompanied by pain and increased sensibility." We rejoice to find more than usual stress laid upon "disorder of function"—a state in inflammation left by many authors rather to be understood than described, always existing and persistent to a greater or less degree through the progress of the disease, and often the first recognisable symptom.

In describing the Constitutional Symptoms, Mr. Miller adopts the opinion, that the buffed and cupped condition of the blood depends upon changes induced in the blood itself by the inflammatory process, an increase in the quantity, and tendency to aggregation of the fibrine and lymph-globules. We are hardly satisfied with the proof of the necessity of the positive alteration in the vital and physical characters of the blood, believing that the physical laws, when the vital or physiological are *slowly* withdrawn, are sufficient to account for the complete attraction of similar globules of the blood towards their own kind, and thus to perfect that separation which the more rapid coagulation of the same blood from a healthy individual prevents; in fact, possessing a higher amount of vitality, in consequence of the inflammatory fever, or over-action and innervation from any cause, it parts with its life, and consequently coagulates, more slowly, allowing time for the completeness of the separation of its constituents. Our author makes some short but pertinent observations regarding the character of the pulse in inflammation of different organs; a condition, we fear, too much neglected in practice, though of the utmost importance, and generally taught in the medical schools. Mr. Miller very wisely guards the practitioner against officiously interfering to arrest a *critical* evacuation, when inflammation is likely to subside in consequence, as a profuse and sustained perspiration; a diarrhœa; a copious flow of "urine, more aqueous, less saline, at each evacuation less and less coloured, and, on cooling, letting down a large quantity of lateritious sediment, composed of urate of ammonia, more or less coloured by purpuric acid; or a discharge of blood from the rectum, the urethra, the mouth, the nose, according to the part affected." We allude to these critical discharges, not that we think they are frequent, but that when they occur, as undoubtedly they do, they are not sufficiently regarded, and malapraxis is often the consequence.

The results of the inflammatory process are elucidated by an "accompanying diagram, which, though both rude and fanciful, may assist to make them more plain. It will also illustrate the opinion held as to the gradual formation of the true inflammatory crisis." (P. 76.) These results are arranged under the heads of—1. Resolution, including Delitescence, and Metastasis, relative to which we have some good observations. 2. Excessive Deposit, by Exudation through the Vascular Coats yet entire; of Serum; of Plastic Fibrin; the organization of the latter requires a lower degree of action than true inflammation, as is corroborated by Mr. Dalrymple's paper. "But to all fibrin so organized, a general rule seems to be applicable, viz. that it is of low or imperfect organism, and, by consequence, liable to destruction in one of two ways; either by simple absorption, or by secondary action advancing to suppuration and ulceration. This is favourable; as regards the discussion or disintegration of simple enlargements of inflammatory origin, unfavourable, as regards reparations of solutions of continuity; and hence it is that the cicatrix by granulation —a process always preceded by true inflammation—is often undone, and the wound made gaping as before, while union by adhesion, or by the slow modelling process into whose composition true inflammation does not and cannot enter, remains firm and enduring." 3. Inflammatory Hæmorrhage and Extravasation. 4. Suppuration; many excellent observations occur upon the characters and formation of Pus; and upon the Constitutional Irritation so frequently accompanying Suppuration. "Pus may be regarded as a changed condition of the liquor sanguinis, consisting of globules, and small molecular particles, more or less numerous, contained in a thin serum. The serum is analogous to that of liquor sanguinis, the globules and molecules to its fibrin; indeed, there is every reason to believe that the globules are actually fibrin in a degenerated form. The formation may be either intravascular or extravascular." Our author thus holds the opinion of Sir E. Home, that pus is fibrin and serum changed in their characters, often after they have left the blood-vessels, sometimes previously to their escape. 5. Ulceration. Mr. Miller combats the Hunterian theory, that ulceration was the exclusive work of the absorbents. "Without denying that absorption, by both lymphatics and veins, goes on to some extent during ulceration, and that part of the destructive process may be so produced—yet there is every reason to believe that the major and more important part is effected, independently of that class of vessels." "The steps of the process are—1. True inflammation, with suppuration; 2. Softening of the truly inflamed part; 3. Its reduction towards a fluid form—a vital act more or less complete; 4. Disintegration, or death and detachment in minute portions, or molecules; 5. Mixture with pus, and removal in one common discharge. Pervading this process, there is some absorption; but the amount of that action is not only inadequate to effect the change, but is even below the ordinary standard of health." This account of ulceration differs widely from the ancient doctrines of corrosion, and solution. 6. Mortification. Mr. Miller gives an opposite account to that of the late Sir Astley Cooper, of the comparative extent to which the various structures of the limb will slough; the latter great surgeon, describing the separation to take place higher in the muscular, then the fibrous, and, lastly, in the osseous textures, than in the skin, thus forming a good stump with ample integument; whereas, our author says, "But Nature's amputation is, unfortunately, a reverse of the ordinary operation; producing a stump conical, and otherwise but ill-fashioned for useful purposes. The surgeon is therefore called upon to interfere in most cases, to modify the arrangement, and secure division of the bone or bones at a higher point." The constitutional symptoms of mortification are given in an extract from Mr. Travers' philosophical work on Inflammation.

With the view of preventing the accession of inflammation immediately after the removal of the exciting cause, Mr. Miller prefers the steady application of cold and moisture to hot water or steam, which, though successful in subduing "the nervous excitement, or breaking off the first link in the chain, may be too great a stimulant to the second step of the initiatory process." Unhappily the medical practitioner rarely has the opportunity of applying his remedies during the early "incubation" of the inflammatory process. We cordially consent to the observations respecting the vast importance of the removal of the cause in the treatment of inflammation; no surgeon will deny its propriety; yet how frequently is it neglected; "cases are on record of eyes having become pearly white and sightless, notwithstanding the induction of anæmia, dropsy, and mercurial disease—premature age and infirmity—by the attempts to save; all the while some small particle of foreign matter lodging undisturbed, and probably unsuspected, in the lining of the upper eyelid, whose simple removal might have saved both eye and system to the patient, as well as credit and conscience for the practitioner."

Some practical and excellent remarks on General and Local Blood-letting follow; the *tolerance* of bleeding and other powerful remedies induced in the system by disease should ever be borne in mind, and hence the extent of the antiphlogistic and sedative treatment is to be regulated by its effects, not by its amount: this tolerance, however, being much influenced by age, sex, temperament, acquired or cognate debility, &c. &c. We are gratified to find a full opiate recommended in the asthenic or nervous re-action from bleeding, having experienced great advantage from its administration; and, further on, that it is proposed to use it more extensively for its own direct influence, as well as to modify the effects of bleeding and of mercury. We have no doubt that the late Dr. Armstrong was justified in his predilection for opium in the treatment of inflammation, and we fear that he urged the use of the remedy upon the profession with too little success; in some late instances of peritonitis after the extirpation of the ovarian cyst, it has been most beneficially employed, even without bleeding, and where the loss of blood was contra-indicated. Alluding to the occasional difficulty in reaching a vein for the purpose of bleeding in the usual sites, our author says, "it is to be remembered that a sufficient vein—the cephalic, is always to be found by a slight and sure incision, placed in the interspace between the deltoid and the clavicular portion of the pectoralis major muscles." "Hæmostasis, or temporary arrest of a portion of the blood, apart from the general circulation," is here quoted from the Maryland Medical and Surgical Journal, as having been proposed "as an occasional, or perhaps even frequent substitute for blood-letting; or, at all events, as an useful auxiliary. The blood of a limb, or of limbs, may be readily retained therein for some time, by deligation sufficient to arrest the venous return; and this may possibly have the effect of relieving the general circulation; the sluices being always slowly opened, so as to admit the gradual escape of the pent-up fluid. Such procedure is sufficiently ingenious, and not unpromising in theory; but it requires attestation by experience ere it can be recommended in practice."

Some useful remarks upon local blood-letting occur; among the precautions connected with the application of leeches, we would allude to the occasional apparent poisoning by the leech-bites; we say apparent, as it may still be doubtful whether all the cases of irritation and ulceration following the bites may not be referred to the condition of the skin to which the creatures have been applied: yet as most, and probably all, the animal fluids possess a tendency to excite disease in the recipient structure when predisposed, it is not too much to attribute many of the instances of severe irritation, inflammation and its worse consequences, to the direct poisonous nature of the secretion of the leech. Mr. Miller's experience differs from our own in the conception that opium is as readily absorbed from the rectum as when administered by the mouth. We are quite aware that dangerous, even fatal, narcotism has been produced, when opium in a fluid state has been administered by the rectum in doses similar to those given by the mouth; these cases we cannot but regard as rather exceptions to the general rule, and not as establishing it, and moreover they have usually occurred in young children. We would not, however, weaken our author's warning, that "the dose should be the same, certainly not greater," but merely guard against disappointment, if the effect expected should not follow, and justify a quicker repetition of the remedy when applied by the rectum than when by the mouth.

The observations upon "the unsettled point whether heat or cold be the preferable application to an inflamed part," are very judicious, and, it is to be hoped, will assist in deciding this question, more important than may at first sight appear. Mr. Miller "believes heat and cold to be both valuable antiphlogistics, but that each has its own appropriate period for use, and that either, employed out of its proper time and place, will invariably do harm. The virtue of cold is chiefly as a prophylactic, diligently and carefully employed during the period of incubation." "Cold is found to be of use at both extremes of the inflammatory process—just before its accession, and subsequently to thorough recession; but, during the actual existence of the action, it is inapplicable." "*Heat* and *moisture*, plainly less suitable than continuous cold during incubation, are as plainly preferable during the inflammatory process." We must refer to the work itself for the precautions in applying these remedies. Our experience, and we expect the general experience of surgeons, fully confirm the author's laudations of nitrate of silver as an antiphlogistic, as well as a counter-irritant when more severely applied. It is wisely stated, under the head of counter-irritation, that "there is no more valuable remedial agent; none more frequently employed with the best results; but it must be rightly placed and timed; not too soon, nor too near, nor yet too far away." This class of remedies is arranged under three heads, Rubefaciants, Vesicants, and Pyogenic Counter-irritants: the latter "prove highly evacuant, by establishing from the artificially inflamed surface a more or less copious discharge of pus—that is, of the most important part of the blood for nutritive purposes, whether normal or perverted—its liquor sanguinis." Though Mr. Miller regrets "to find the indiscriminate and frequent use of the actual cautery threatening to return," he recommends its use in some affections as very superior to all other means, as "in advancing destruction of texture in the bones of joints," in chronic affection of some of the internal organs also—the kidneys for example." We cordially concur in the remark, that the cases which absolutely require the use of the actual cautery, are comparatively limited as to occurrence; "and for these, in the name of humanity, common sense, and propriety, let it be reserved."

Congestion, the second division of the "Perverted Vascular Action," is described as of two forms, Active and Passive. The first "may be a mere preliminary to the true inflammation; or it may persist as the minor grade, constituting a disease of itself." "Passive Congestion may follow on an imperfect resolution of the active form;" "or it may be original, unpreceded by excitement." "In the active form, the arteries and capillaries of the part are chiefly implicated—dilated, yet carrying on a tolerably vigorous circulation; in the passive, the capillaries and veins are mainly concerned—dilated, but with a circulation much retarded and depressed." The results of Congestion, more especially of the Passive State, are Resolution, Hæmorrhage, Serous Effusion, Inflammation.

The second Chapter treats briefly of Irritation, "Perverted Action of the Nervous System;" a subject hardly of second importance to Inflammation and its results. In describing the symptoms of "The Shock of Injury," Mr. Miller states that, "often the sphincters are relaxed, fæces and urine seeming to pass off involuntarily;" is this the condition as the immediate effect of the shock, or of the suspension or diminution of the action of the nervous system? In Lesion of the Brain, or of the Spinal Marrow, suspending its function, it is well known that, while the fæces pass off involuntarily, the urine is retained; a fact readily accounted for; when however the elastic tissues, as well as the muscles, are influenced by the general lowering of vitality, then, the retentive power of the bladder being diminished or lost, the urine dribbles away though the bladder may still be full, a circumstance not unfrequently mistaken by the inexperienced for incontinence.

"The Shock of an injury may be considered practically as of two kinds—mental and corporeal." "There are many cases in which both forms of shock are more or less combined. For example, a man may be mortally wounded by an unexpected and unseen foe; the shock of the injury will be great, although entirely corporeal in its origin. A second may receive only a scratch, while he expected nothing but instant death; the shock will be serious, and may indeed amount to actual syncope; yet it is purely mental. A third may sustain a serious injury, from an assailant both seen and feared; the shock will probably be intense; mental and corporeal impression both contributing towards the lowering result. In such cases as the last, it is practically useful to ascertain, if possible, in what proportions the combination has probably occurred."

We have made this extract from the conviction of the great importance of this subject, and the fear that surgeons not unfrequently commit errors in practice for the want of its due study: the works on Military Surgery, in particular, teem with cases of moral and physical shock so extraordinary as to appear miraculous to the uninformed mind. Some valuable cautions are given against too early bleeding, and against too early stimulating; against interfering with Nature's "beautiful adaptation of circumstances to the attainment of an important and salutary event." Among the stimulants our author does not mention the hot-air-bath, a remedy more effective and more manageable than internal stimulants, and is immediately applicable in every poor man's cottage that contains blankets, bent rods or sticks, a large beer-funnel, and spirit of any kind.

In considering the "Perverted Action of the Absorbents," it is stated that "it is plain that the anormal state (of absorption) may depend either on excess of absorption, or on deficiency of arterial supply;" a conclusion not so plain as our author would wish it to appear. It is quite possible that the action of the absorbents may be but little, if at all, interfered with, and that the lessened nutrition may be the main, if not the sole, cause of undue absorption, as it is so called; this appears to be the more probable in "continuous absorption" from pressure, occurring without inflammation, and consequently without secretion of any kind, as the absorbent vessels are capable of resisting a greater degree of force than the blood-vessels, and particularly than the capillaries; hence, pressure is so much the more likely to affect mechanically the latter, than to stimulate (how ?) the former. Here are some ambiguous observations on the contrast of absorption with granulation.

The fourth Chapter contains an account of Abscess, with some remarks on Scrofula. Mr. Miller states the Pyogenic Membrane (the lining of an Abscess) "to be endowed with very considerable powers of secretion; but as an absorbent surface comparatively feeble." A circumstance which seems to vary, as pus is known to be occasionally very quickly removed by absorption; but, as a general remark, the above is doubtless correct. He again notices the important fact "that the pus-globule, when extravascular and complete, is of comparatively large size, not soluble in its own serum, and therefore but little amenable to ordinary absorption; the serous portion may be taken up readily enough, but the solid probably remains little affected." Our author does not attempt to account for the process of *pointing;* as the abscess always proceeds in that direction offering the least resistance, whether towards the skin, or towards a mucous outlet, we are inclined to attribute important influence to the pressure of the matter from within the cyst; and probably by interfering with the nutrition of the parietes in that direction. We cannot but conceive that many of the phenomena we are accustomed to regard as vital, are much much more controlled and directed by mechanical operations than is usually supposed. We entirely consent to the remarks on the propriety of early opening an acute abscess; and regret that in some medical schools the opposite treatment is taught; it is quite certain that Nature is sometimes equal to the task of removing the pus by absorption, but this is so rarely met with, that, in the treatment, a judicious surgeon would not reckon upon it; further, in the instances which have fallen under our notice of successful attempts to produce absorption, and thus spare the lancet, the injurious effects of the remedies upon the system at large were any thing but encouraging to a repetition of the practice. When for the sake of removing attenuated skin, or to destroy obstinate glandular enlargement, or in consequence of the patient's objection to the knife, the evacuation is performed by the caustic, our author prefers the "potassa fusa pressed firmly on the part, till the abscess is entered—moved laterally also, if need be, to destroy integument—or pushed deeply, to break up glandular enlargement."

Mr. Miller says "it is probable that, in the truly chronic abscess, enlargement of the suppurated space never occurs by ulceration, but is effected merely by condensation, and by interstitial and continuous absorption of the surrounding parts; unless, indeed, acute accession supervene." An excellent mode of evacuating the contents of a large chronic abscess is described; the ordinary manner of opening these large cysts is often productive of considerable danger by the supervention of acute inflammation of the sac; though at other times the moderate excitement occasioned is productive of benefit.

"An abscess is said to be diffuse, when the suppuration is not surrounded and limited by plastic fibrinous effusion; and when consequently the pus—in such circumstances of a thin, apparently unhealthy, and probably acrid nature—is so soon as formed readily infiltrated into the surrounding texture, open and unprotected;" is this correct? is it not rather that the cyst formed in the usual manner, but in the chronic form, is thin and attenuated, and in consequence of pressure of the pus or of external accident, it gives way more or less suddenly, and the fluid extravasates into the surrounding cellular tissue? or that, in some cases, the sac becomes altogether absorbed, and hence, not being discovered upon examination, is supposed not to have existed? this is a more probable account than Hunter's of his "Collections of Matter without Inflammation:" we grant that suppuration, may occur so rapidly, in cases where the exciting cause is extreme, as in extravasation of urine, as not to afford time for the formation of the cyst; but these are instances of the most acute suppuration, and not very frequently occurring, as the excitement is usually too great for suppuration, and mortification results.

We are favoured with some good, though brief, remarks upon Scrofula; Mr. Miller adopts the views of Carpenter, Bennett and others, and now generally received, that this baneful disease is one of perverted nutrition, the albumen prepared by digestion is not converted into fibrin, "hence albuminous tubercle is deposited in the interstices of the tissues, instead of these tissues being themselves regenerated by organizable fibrin." "The inflammatory process, occurring in one of a strumous habit, may be accompanied by rapid and extensive deposit of tubercle instead of the ordinary plastic exudation."

The fifth Chapter, under the heading Ulceration, treats of Granulation, Cicatrization, and the different forms of Ulcers: the observations on Granulation would almost lead to the inference, that the lost texture was truly reproduced, a supposition with which we cannot concur, but would agree with the quotation from Mr. Travers, in speaking of Cicatrization, that "the new formation is only a copy, and like all copies inferior to the original:" we doubt whether any texture is really reformed by granulation, the organized fibrin, however, constitutes an admirable substitute in most instances, but still a substitute.—Mr. Miller allows the possibility of cicatrization to take place from granulations themselves independent of the original skin, but considers it a very rare circumstance.

We would impress upon the medical student the moral bearing of the introductory remarks to Ulcers, the very opprobria of surgery, more important than most diseases that fall under the notice of the surgeon,—in consequence of their great frequency, general difficulty of cure, affecting, for the most part, the legs of the labourer, who cannot afford to maintain the rest and position often so necessary to cure—yet frequently neglected in their study, and passed by as of secondary importance.

> "The following classification will be found to include the great majority of ulcers. Under one or other of the varieties every example may be arranged; or, if the exact type be not there, it will be found somewhere intermediate, and easily deducible therefrom. 1. The Simple Purulent, or Healthy Sore. 2. The Weak. 3. The Scrofulous. 4. The Indolent. 5. The Irritable. 6. The Inflamed. 7. The Sloughing. 8. The Phagedænic. 9. The Sloughing Phagedæna."

Mr. Miller often alludes to the efficacy of the water-dressing, medicated or not, in the treatment of some forms of ulcer, and especially advocates the free use of "potassa fusa in solid substance," to remove the unhealthy infiltrated cellular tissue in the scrofulous sore, "so as to destroy thoroughly not only the cellular tissue where tuberculated, but also the integument where thinned, blue, undermined, and obviously incapable of recovery." "It is, avowedly, a painful process, but most effectual; indeed, according to my experience, altogether indispensable towards obtaining a satisfactory cure." Thus is strongly recommended the practice some years ago urged by Mr. Lloyd. The Indolent Ulcer, most frequently occurring in the lower extremities of the old and unhealthy, who, at the same time that they have borne more than their share of this world's labour, have enjoyed less than their fair proportion of this life's comfort, or even nourishment; support and pressure by bands of plaster and rollers, introduced by the late Mr. Baynton of Bristol, are recommended as the best means of restoring a more healthful action, and tending to granulation: but perhaps hardly sufficient stress is laid upon this indispensable treatment; which, even when incapable of inducing the curative process, is often sufficient to retard the progress of the disease, and to render a limb, though ulcerated, useful for the purposes of even laborious exertion, and free from pain and distress.

In the treatment of the Sloughing, Phagedænic, and Sloughing-Phagedænic Sore, Mr. Miller advocates the use of "severe and active remedies —escharotics—at the outset, in order to cut short the disease, and along with the suitable constitutional treatment—to change the character of the sore into the healing type," in preference to the "lenient measures—poulticing, rest, and expectancy." Nitric acid undiluted is the remedy our author freely applies; when the eschar has separated, if the slightest appearance of phagedæna continues, the escharotic is to be renewed; he considers, however, that the nitrate of mercury is preferable in the re-applications, "not as a more efficient escharotic, but as a more successful *alterative* of the nature of the sore." It is stated "that this class of sores is communicable by contagion;" is this really the fact? that many, perhaps most, sloughing ulcers are specific, and consequently contagious we will admit, but are hardly prepared to receive the above dogma, as respects sores arising from common causes, and assuming the phagedænic character, in consequence of a debilitated or strumous constitution, or from untoward external circumstances. The Varicose, and the Menstrual Ulcer, of some authors, are regarded as conditions or peculiarities, which may attend every variety of sore.

Mortification occupies the sixth Chapter. In treating of the causes of this extreme result of Inflammation, Mr. Miller has an interesting extract or two from Baron Larrey's famous work, shewing that mortification is, in by far the majority of instances, the immediate effect of re-action from severe cold, and not the effect of cold, although long endured, and even fifteen degrees below zero of Reaumur's thermometer. We are gratified to find allusion made to the influence of mercury, as predisposing to sloughing; and we incline to the belief as producing the immediately exciting cause in many cases: we would that this fact were more constantly in the minds of those practitioners who recklessly abuse this valuable medical agent. Mr. Miller does not regard Arteritis, terminating in consolidation of the vessel, as a frequent cause of the death of a limb, large or small; and, although he would not deny its possibility, seems to regard it more reasonable to attribute it to the diseased action "having invaded, not the arterial tissue alone, but the whole part." Hardly feeling justified by experience to regard, with Dupuytren, adhesive inflammation of the arteries as the most common cause of gangrene in the extremities, we are quite convinced it is far from infrequent; we believe that this cause was first pointed out by the late Mr. Grainger, Sen. of Birmingham, who detected it in a soldier of his regiment, when quartered in Ireland, further investigations brought more proofs; since which time they have been multiplied; the most interesting case of the kind which has fallen under our notice being that of Mr. Crisp, in which the great trunks of both the upper and lower extremities were more or less consolidated; one, if not two, completely so, in a young girl, to all appearance healthy, with this exception, and that of the resulting mortification. Our experience does not coincide with the author's that Senile Gangrene "is most liable to occur in males, of the higher ranks, and who have indulged freely and habitually in the pleasures of the table," as we have witnessed the disease much more frequently in the lower orders, who have freely indulged in spirituous liquors, and have been much exposed to wet and cold, being now, in their old age, badly fed and badly clothed; the diseases, however, to which the two extremes of social rank are predisposed are very similar, for the habits, when an unchecked license is given to sensuality, though apparently the very reverse from each other, tend to the same unhappy result.

Mr. Miller very justly guards his readers against false diagnosis, against mistaking the serous vesicles of bruise for the phlyctenæ of mortification, an error not likely to occur to the well-informed surgeon, but which has happened to the less accomplished. Many cases of mortification from pressure, or *bed-sores*, would be avoided by the more constant use of "that admirable contrivance, the hydrostatic bed, by which the labour of support is equally distributed on every part of the surface;" the reddened and painful parts being at the same time "pencilled over by nitrate of silver, either in substance or in solution, so as merely to blacken the integument, carefully avoiding the vesicating effect." The old and empirical practice of rubbing the frost-bitten foot with snow, while the patient and part are yet in the open air, and upon the success of which the disciples of Hahnemann partly found, though falsely, their doctrines, "ensures the reaction being gradual, slow, and safe." We are favoured with some good remarks upon the middle course of general treatment, avoiding stimulation on the one hand, and spoliation on the other. Among the local, as well as the general remedies for mortification, Mr. Miller, very properly, denies there are such remedies as antiseptics; those applications which were so imagined are all stimulants, and for the most part injurious as such; the chlorides are useful when applied to the decomposing parts, as correctives of fætor."

Mr. Miller concludes the important question of Amputation in mortification, concerning which so many contradictory opinions have been entertained, with this admirable summary:—

"Thus then, when gangrene is acute and humid, dependent on an external cause, and unconnected with a previously existing failing of system or organic change in the general limb, we amputate, if at all, during the progress of the disease, without waiting for a line of demarcation. When it is chronic and dry, dependent on an internal cause only, or on internal more than on external causes, and connected with failing of both general and local vital power, we wait for the line of demarcation, watch the progress of separation—cautiously supporting the system meanwhile—and when detachment is far advanced, we interfere merely to facilitate and modify its completion; we amputate in the line of separation. When gangrene is the result of one particular external cause, cold, we await the line of demarcation; and, so soon as that has been fairly formed, we amputate either there, or above, according as circumstances may seem to require."

Having noticed at considerable length the Section containing inflammation and its results, we may be excused for making brief allusion to the remainder of Mr. Miller's book; the more so, as complete treatises upon the subjects are not to be expected in a work professedly upon the Principles of Surgery.

The seventh Chapter, the first of the second Section, describes Erythema, Erysipelas, Hospital Sore, Furunculus, Anthrax, and Diffuse Cellular Infiltration. Mr. Miller has described five varieties of Erysipelas, the Simple, the Phlegmonous, or Cellulo-cutaneous, the Œdematous, the Bilious, and the Erratic; and in addition, the Hospital Erysipelas. Such division may facilitate the recollection of the forms and degrees the disease may and does assume under peculiar conditions, but beyond that it has a tendency to mislead the student into the expectation that the varieties are different affections. We entirely concur in the general treatment of this troublesome, and often dangerous complaint, and especially in our author's laudations of punctures and incisions judiciously performed, and at a period judiciously chosen. The question of the contagious nature of erysipelas is wisely left open, with the recommendation that every precaution against communication should be taken. We, however, entertain no doubt of its non-contagious character, though in the crowded wards of an hospital, particularly in those appropriated to accidents and operations, it frequently presents an apparent even infectious nature; a circumstance, however, more legitimately accounted for by the general contamination of the atmosphere, and the peculiar disposition of all the patients there domiciled to be affected by this very complaint.

Mr. Miller writes that Hospital Gangrene "is an example of sloughing phagedæna, it may be produced directly by contagion, more indirectly by infection; or it may occur independently of either—from crowding, evil dressing, or noxious atmospheric influence. Mercurialism is especially favorable to its accession. It may either seize on a wound already existing, or appear in a part previously entire." As "hospital erysipelas and hospital sore may be found to co-exist," which we believe to be of frequent occurrence, and that one often runs into the other, are they not both subject to the same laws of origin, and may not the same doubt of the contagious or infectious nature of the one apply with equal propriety to the other? It is stated that "the constitutional symptoms of Carbuncle or Anthrax are asthenic throughout; at first of a simply febrile and bilious character; then shewing typhoid signs:" our experience does not confirm this statement; we have seen carbuncle occur in the over-fed and plethoric, and accompanied by inflammatory symptoms requiring mild antiphlogistics, though the reverse is its ordinary character. Usually, "Diffuse cellular infiltration is connected with the inoculation of a specific virus." The bites of reptiles, stings of insects, and punctures received during dissection, are familiar examples of such exciting causes." "Urine infiltrated into the cellular tissue is certain to light up an asthenic and rapidly destructive inflammation there." It is an interesting fact, to which allusion is not made, that the danger to life in extravasation of urine is proportional to the extent of destruction of the skin, a very extensive sloughing of the cellular membrane may occur, and the recovery be tolerably certain in a previously healthy individual, but if the integument slough to a similar, or even less extent, a fatal issue is to be expected; hence the utmost importance of free and early incisions through the skin.

The "Perverted Vascular Action occurring in Bone," is considered in the eighth Chapter. We prefer Dr. Cumins's arrangement of this important part of pathology to that before us, as being more simple, and more intimately allying the usual changes in the osseous structure with the producing inflammation. Our author treats these diseases under the heads of Periostitis, Ostitis, Suppuration, Absorption, Ulceration of Bone, Caries, Necrosis, Fragilitas Ossium, Mollities Ossium, Rickets.

"Examples are not wanting of the whole skeleton having been involved in periostitis ;" happily such extent of this direful affection is rare, though we have seen, too frequently, the vastness of its frightful ravages. Mr. Miller properly attributes much blame to the abuse of mercury, and says truly, that " the worst cases are those which occur in scrofulous patients, who have suffered from both syphilis and its supposed specific." Let it not however be inferred that we doubt the specific properties of mercury for the cure of syphilis; it is the abuse of this valuable medicine, and its non-regulation in accordance with the exact form of the disease, and more especially with the constitutional peculiarities of the patient, and the circumstances surrounding him, to which we would refer as inducing the various forms of mercurial disease.

Allusion is made to the Periosteum being sometimes the seat of neuralgic affection; it may follow amputation, or slight injury; requiring rest, the endermoid application of nitrate of silver, and the internal use of tonics, &c.

The result of Ostitis " may be suppuration, internal, external, or general; ulceration, simple or carious; local death or necrosis. Or the action not reaching true inflammation, and imperfectly resolving, there may be simply change of structure." The " change of structure" is the hyperostosis of Dr. Cumins, in which the bone is at first softened as well as enlarged, and apparently porous, afterwards condensed and indurated by excessive deposit of earthy matter.

Suppuration originates in the bone or extends to it from the periosteum and forms abscess, internal or external, acute or chronic: the internal abscess being also *diffuse* or *limited*. Mr. Miller, in our opinion judiciously, urges the adoption of free incisions to allow the matter to escape as readily and as easily as possible, and the internal abscess to be opened by the use of the trephine if necessary, as proposed by Sir Benjamin Brodie; by which active treatment necrosis and exfoliation is often prevented, the suppuration limited, and the limb saved. We have described the " Scrofulous or Tubercular Abscess of Bone," and also " General Suppuration of Bone," in which " the abscess is neither external nor internal, but diffuse, pervading the whole thickness of the bone, and invariably acute; the result of intense general ostitis," " in fact the case is one of acute Necrosis."

" Absorption of Bone is more or less connected with perverted vascular action, but altogether independent of true inflammation." It is interstitial or continuous: of the former we have examples in the commonly named " white-swelling," and the wasting of the bones; of the latter, in the effects of pressure from aneurism, abscess, or solid tumors.

Mr. Miller describes two varieties, or rather degrees of Ulceration in Bone; the Ulcer, which is simple and tractable; and the Caries, which is peculiar and difficult of cure. The Ulcer " is the product of true inflammation, as in the analogous condition of the soft tissues." The Caries " is something more than a weak ulcer of bone; it is something less than a malignant or cancerous sore, as it is too often designated." It may be " simple, or of a scrofulous or tubercular character." Our author objects, and with reason, to the indefinite use of this term Caries: it is generally used to imply every ulcer, or even absorption of bone, whether of the skeleton in general, or of the tooth, whether arising from a common or a specific cause, whether terminating in the destruction of the bone, or healing by granulation; we can hardly object to its use as a generic term, though we certainly should if it were intended to convey any peculiarity, or the want of peculiarity, in the disease.

Necrosis is most "frequently the indirect result of injury, the bone perishing by an overpowering inflammation;" it is simple when unaccompanied by any other form of disease; compound if combined with caries, or attendant on fracture. It is also traumatic when arising from injury; idiopathic when originating without any appreciable exciting cause. The extent is very various, hence we have exfoliation, the death of a mere scale of bone; or the cancellated interior may perish alone; or the whole mass may die, forming general necrosis, an unusual circumstance, as the articulating extremities generally escape. Mr. Miller divides the process of Necrosis into stages: first, "the bone, or portion of bone inflames:" second, "the bone dies:" third, "the dead portion is separated from the living;" fourth, "separation of the dead portion is completed." Are not these two stages one and the same? fifth, "the dead portion is extruded;" sixth, "reparation is completed." The important practical fact is generally, but not universally, acted upon, that a bone denuded of its periosteum does not necessarily die; its destruction depending upon the extent of succeeding inflammation. The injurious consequences of interfering with the sequestrum, in all cases, "before it has become loose," cannot be too strongly urged; much evil, we are convinced, is daily produced by the attempts, generally and fortunately too unsuccessful, to pull away the bone before its separation from the healthy texture. We rejoice in the opinion that "amputation is rarely demanded in necrosis; that it is the exception not the rule." The medical philosopher is probably more gratified at the wonderful operations of nature in restoring a necrosed bone, and removing the sequestrum, than in most of the other resources of the vital power; and we regret that we cannot make further observations upon the text before us, or extract more largely; we therefore refer the reader to the work itself, promising him ample reward for his pains.

Fragilitas Ossium; is it quite certain that this affection of the bones really exists? the varying proportion of earthy matter at different periods of life renders the bones more or less brittle, and consequently more liable to fracture, and, without denying that over-deposit of bone-earth may occur, producing disease, we think the examples reported may be more truly referred to other conditions of the bones, as ulcers, common or specific, malignant deposits, softening, &c. &c. The opposite state, Mollities Ossium, or Osteomalakia, is a really formidable disease, happily not of great frequency, yet every surgeon of practice must have witnessed several examples; we have lately seen a case in which every bone of the skeleton appeared to be affected, those of the extremities being bowed according to the pressure, and the pelvis and shoulders being distorted from lying, when the recumbent posture became inevitable.

In describing Rickets, our author says, "the bone is found changed in structure, much in the same way as in fragilitas ossium; but instead of a brittle condition, the result is softness and pliability;" surely this disease is more allied to mollities ossium; it is the softening of bone in the young, one result of a scrofulous diathesis. Mr. Miller recommends light mechanical support as necessary for the spine; this must be applied with great caution if at all; we have not the great dread of taking off the weight from the skeleton by means of the recumbent posture strictly maintained; the pressure is the exciting cause of the deformity, the constitutional deficiency, the cause of the mal-construction of the bones; either cause, if neglected, will operate upon the other, the latter more effectually; and conceiving that both indications might be successfully attended to, we have followed the plan they have suggested, and with satisfactory and gratifying results.

The ninth Chapter is a short dissertation on the all-important Diseases of the Joints, which were at one time all inconveniently included under the designation "White-swelling." Mr. Miller "considers, in succession, the results of the inflammatory process in the different component textures of the joints; 1. In the Synovial Membrane; 2. In the Cartilage; 3. In the Bones. Synovitis is Acute, Chronic, and Scrofulous also Chronic. "The Brown Intractable Degeneration of the Synovial Membrane," Mr. Miller thinks is allied to Carcinoma, and if it be not malignant at the onset, very liable to become so; we would consider this peculiar affection as originating in a scrofulous rather than a carcinomatous tendency, which opinion does not preclude the supposition of it eventually assuming the malignant character under favouring circumstances. It is described as an affection which can only be ameliorated at its very commencement, afterwards the limb, or later, the life, of the individual must be sacrificed. "The Fimbriated Synovial Membrane" is a rare affection, and the history of it is taken from Mr. Liston's Elements of Surgery.

"The Inflammatory Process in the Exterior of Joints," is intended, we suppose, to express inflammation in the various fasciæ and cellular membrane external to the joint, whether arising from common causes or from rheumatism, and often endangering the joint in its result.

"Tophi:" "these are concretions (of Urate of Soda) connected with the extreme articulations, more particularly of the fingers." Although these deposits are alluded to here in connection with the diseases of the joints, they are far from being peculiar to the articulations, and, when they are found in their vicinity, we conceive them to be symptomatic of the general rheumatic or gouty diathesis, and are there located in consequence of the fibrous tissue, to whioh structure they appear to be confined; if one part of the body be especially liable to these concretions, we are inclined, from our experience, to name the Fascia Lata; we have lately witnessed the unusual case of a child eleven years old suffering from acute rheumatism with deposits of Urate of Soda in the fascia of various parts, the first and largest and most numerous being in the back part of the scalp or tendon of the occipito-frontalis, then in the forehead, arms, legs, feet, &c. as well as near the large joints; there was, and still exists, deposit, perhaps of a similar character, in the mitral valve; the external tophi having been absorbed furnishes an increased hope that a similar result may occur in the heart. Mr. Miller, however, speaks of them as incapable of being absorbed, which we conceive is opposed to general experience. Benzoic Acid has lately been proposed as an internal remedy, with the view "of converting the uric acid into the hippuric acid," the hippurates being comparatively soluble.

The history of "Destruction of Cartilage" is valuable; it is arranged as 1. "Simple Destruction or Ulceration." 2. "Scrofulous Ulceration of Articular Cartilage." Cartilage is also subject to "Hypertrophy" from over-nutrition; and to "Atrophy," the consequence of over-pressure, hence chiefly met with in old persons. "Porcellanous Deposit" may be the result of ulcer in the cartilage. "Osseous Deposit exterior to the Articulation," results from rheumatism, especially affecting the periosteum. "Interstitial Absorption of Bone implicating the Joint," is a frequent occurrence of great importance, constantly predisposing to fracture, and often mistaken for it: it is the effect of old age, especially in the hip and shoulder joints of females consequent upon the well-known anatomical peculiarities. Mr. Miller describes three modes by which "Loose Cartilages (?) in the Joint" may be formed, a problem, perhaps, not yet satisfactorily solved. We have detailed the improved "subcutaneous and valvular punctures" into the synovial capsule for their removal, as proposed by Mr. Syme, Mr. Goyrand, and also by Dr. Duncan.

"Articular Ulcer," and "Articular Caries" are alluded to very briefly; for the latter, the "Re-section of Joints" is occasionally demanded. We may be permitted this opportunity of congratulating the profession and the public for this important addition in the present day to our chirurgical appliances; by which not only a limb, but a useful one, may occasionally be preserved; we must, at the same time, regret that this improvement has been subjected, like others, to the customary abuse in practice.

"Anchylosis is said to be of different kinds." 1. Osseous or Complete. 2. Ligamentous. 3. Spurious, occasioned by extensive fibrinous deposit exterior to both joint and ligaments. "Neuralgia of Joints," and "Wounds of Joints," conclude this part of our author's subject; with slight allusion to "Affections of Bursæ," and "Affections of Thecæ," including "Bursitis, Acute and Chronic; loose bodies therein contained, and Ganglion."

The tenth Chapter treats of the interesting "Diseases of the Arteries." Arteritis is described as Acute and Chronic; the former subdivided into the "Spreading" and the "Limited." "The Calcareous Degeneration" is very different from the state of inflammation.

"Aneurism. By this term is meant a pulsating tumor; composed of a cyst, which is filled with blood, partly fluid, partly coagulated, and whose cavity communicates with the arterial canal." This definition is not unexceptional, nor does it appear to be an improvement upon that of the late Sir Astley Cooper. Our time and space will permit us only to enumerate the divisions of this interesting disease adopted by Mr. Miller, who still describes it as True and False, terms which, having been adopted before the anatomy of aneurism was really understood, were intended to imply what did not occur; a circumstance, however, of little importance, if the present definition be always understood. True Aneurism is "the result of disease; the tumor being formed by dilatation, or by either rupture of the coats or their ulceration from within, or by a combination of both circumstances; and the cyst, consisting of one or more of the arterial coats, yet undivided." The term *false,* on the contrary, denotes those aneurisms, in which the arterial tunics have been wholly divided, either by wound, or by ulceration from without, and form no part of the aneurismal cyst.

These constitute the minority, the former the majority, of the cases of aneurism." We entertain the opinion, that in many cases of the so-called True Aneurism, the whole of the arterial tissue has been removed, the cyst being entirely newly-formed from the surrounding texture and organized fibrin. The True Aneurism is formed "by dilatation," by "dilatation and rupture," by "rupture." The varieties are the Dissecting and Pedunculated, as also limited or diffuse. False Aneurism may result from a direct wound; from laceration; or from ulceration from without, as from an abscess; as an example of the last mode of formation, Mr. Liston's case is alluded to; yet much doubt exists in the minds of many surgeons as to the correctness of the diagnosis in this case. Many excellent, and somewhat lengthened, observations are made upon the subject of Aneurism, and particularly upon its treatment; we are glad to find due honour given, as it always ought to be when considering this disease, to John Hunter. Mr. Miller here describes "Aneurism by Anastomosis; Vascular or Erectile Tumor," "the Nævus Maternus," although he says it "might have been classed with tumors," we think it would have been much more correct to have so considered it; there is an Anastomosing Aneurism, in which several arteries shoot into and supply a sac, previously formed upon a vessel by injury of that vessel: a condition frequently confused with the nævus, in consequence of the same term being often applied to the two affections.

"Affections of Veins" compose the eleventh Chapter; a subject only of less importance than diseases of the arteries, because their more serious affections are less frequent. They consist of Phlebitis, Varix, and Entrance of Air into the Veins. Phlebitis is described as Fibrinous, and as Suppurative, the latter being limited and diffuse. The diffuse Suppurative Phlebitis being almost certainly fatal, the pus poisoning the whole mass of blood, and forming "purulent depôts," most frequently situated in the lungs. The cure for Varix is Palliative or Radical; our author appears to prefer the twisted suture, the needle passed under the vein, and the ligature over it. Every mode of positively arresting the circulation through the vein, and every attempt to obliterate or destroy it, is attended with danger: we have derived much success from freely blistering the integument over the distended vein or veins; the effect, though expected to be palliative, has occasionally been radical. "Entrance of Air into Veins" is truly an alarming casualty, generally occurring in wounds when a vein of considerable size remains open, or is "canalized."

The twelfth Chapter is occupied by Hæmorrhage, Arterial and Venous, and the Hæmorrhagic Diathesis. The means of arresting bleeding are termed "hæmostatics," which are natural or surgical; the former are most interesting to him who delights in recognizing the astonishing resources of Nature. The surgical means, so important in their prompt application, are here arranged under the heads of—1. Pressure, including the use of the Tourniquet; 2. Position; 3. Cold; 4. Styptics; 5. Escharotics, actual and potential; 6. Plugging; 7. Ligature, "the most sure and satisfactory;" Mr. Miller considers the division of the inner coat of the artery to be necessary in successful deligation; a circumstance which admits of considerable doubt; certain is it that the obliteration of the artery will not be prevented thereby, and most probably it will be favored.

8. Torsion; 9. Nauseants and General Treatment; 10. Syncope. Our author entertains a salutary dread of placing a ligature on a vein, and we concur in the propriety of avoiding such procedure if possible, but we must not risk a greater danger to shun the phlebitis, which most probably will not occur, if the vein be previously healthy: we have seen the ligature applied to the femoral, in consequence of active bleeding after amputation, separate without the slightest inconvenience, even earlier than that from the artery. The hæmorrhagic diathesis is described as hereditary, with "many points of resemblance to both the scrofulous and the scorbutic," its cause being twofold, a morbid condition of the blood, and also of the capillaries.

Angeio-leucitis, or Inflammation of the Lymphatics, with "Inflammatory Swelling of the Lymphatic Ganglia, and Glandular Tumors," compose the thirteenth chapter. The fourteenth chapter contains very brief observations on Neuritis; and remarks, perhaps too much curtailed, on the all-important subject of Neuralgia; the less to be regretted, in consequence of its connexion with Irritation, previously treated of.

The third section of the book, under the heading "Perverted Nutrition," contains in one chapter a dissertation on Tumors. Mr. Miller wisely states, "It is impossible to construct a classification which shall embrace every tumor. We attempt only that which may include the majority; arranging them, also, in a form at once convenient for description, and suitable for enforcement of the practical details of treatment." He then arranges them into "Tumors of the Soft Parts, and Tumors of Bones." The former being of two kinds—*Solid* and *Encysted.* "The solid tumors, again, are *Simple* and *Malignant*. 1. In the former class are the *Simple Sarcoma,* the *Adipose,* the *Fibrous,* the *Cartilaginous,* the *Calcareous,* the *Osseous,* the *Cysto-sarcoma.* 2. There is a tumor locally simple, but accompanied with, and dependent on, a constitutional vice; the *Tubercular.* 3. The malignant are, "the *Medullary, Carcinoma, Melanosis,* and *Fungus Hæmatodes.*" These are alluded to separately, and the malignant somewhat in detail. "The tubercular or scrofulous tumor may be regarded as occupying a middle place between the simple and malignant formations." "The constitutional vice is that of scrofula; the peculiar deposit is of tubercle." Mr. Miller distinguishes Fungus Hæmatodes from the Medullary Sarcoma; and certainly the ordinary characters of each are widely different, however frequently the section of either may disclose portions of the tumor very nearly resembling the other. We have some good remarks upon these diseased growths, and upon carcinoma in particular. They are concluded by "some general observations on their removal by the knife." Under "Tumors of particular Textures," are described, those of the Integument; those of the Mucous Membrane, the Polypi, which are—1. The Simple Mucous; 2. The Cysto-mucous; 3. The Fibrous; 4. The Medullary. Sometimes, but rarely, the structure is carcinomatous. Then follow tumors of the nerves, Neuromata. "Tumors of Bone," like those of the soft parts, are simple and malignant, analogous and heterologous. "The great majority, if not all, are included in the following classification. Exostosis, Osteoma, Enchondroma, simple; Osteosarcoma, at first simple, but tending to degeneration and malignancy; Osteocephaloma, Osteo-carcinoma, malignant; Osteo-aneurism, troublesome and tending to disaster, sometimes associated with malignant structure; Spina Ventosa, analogous to the encysted tumor of the soft parts." Several of these bony tumors have varieties, as the Exostosis, Enchondroma, &c.

The fourth, and last, section is on Injuries; the first chapter of which, the sixteenth of the book, treats of Wounds, which are Incised, Contused, Lacerated, Punctured, Poisoned, and Gun-shot. Some judicious remarks are made upon the healing and treatment of Incised Wounds. Mr. Miller strongly urges delay in closing the wound till all hæmorrhage has ceased, and the cut-surfaces are glazed by the exudation of the liquor sanguinis, the solid part remaining on the wound, the serum trickling away. The cold-water dressing, as proposed by "the old man of Cos," he recommends till the wound is closed, and then the isinglass plaster introduced by Mr. Liston. We would direct the reader's attention to the account of poisoned wounds, including Bites of Serpents, and Hydrophobia. The average who fall victims to hydrophobia of the number of people bitten by the same rabid animal, is supposed to be one in twenty; a very hopeful assurance for the unfortunate; recovery from the disease, rarely, or perhaps never, happening: the average period of incubation "may be said to range between five and ten weeks." Without contradicting this supposition, we would state that we have had undoubted evidence of the virus laying dormant for a year, and in one instance even more. The human subject is also sometimes inoculated with the poison of glanders or farcy, Equinia; as well as with the "Malignant Pustule or Vesicle," "from animals affected with typhoid disease." The chapter concludes with a description of Tetanus, "the morbid appearances of which, found after death, are similar to those in hydrophobia." "The spinal cord usually evinces manifest congestion, both in itself and in its membranes; more especially at the origins of the nerves: with the serum preternaturally and considerably increased." Is this condition really USUAL? Does it not more frequently happen that the spinal cord, and indeed all the other organs, present morbid appearances which can be more readily accounted for as the consequences, rather than as the cause, of this formidable and hitherto mysterious malady?

Burns and Scalds compose the seventeenth chapter. Our author describes six degrees in which the injured part may be affected by the all-powerful agency of heat. The treatment of these extensive injuries, often so empirically conducted, is very simply and sensibly laid down upon the ordinary surgical regulations.

Chapter eighteenth, Effects of Cold, Frost-bite, and Chilblain.

In the nineteenth chapter, Fractures of the Bones are discussed, with their kinds and characters; their predispositions and symptoms; prognosis and mode of union; their treatment, including reduction and retention; in which our author does not give that unqualified assent to the gum and starch bandages for which many surgeons now contend; our experience, however, of their usefulness leads us to anticipate their almost general use; lastly, we have the important treatment of a false joint from an ununited or a disunited fracture. Dislocation follows in the twentieth chapter, with its causes, symptoms, consequences, and treatment. The two concluding chapters treat very briefly of "Sprain, and Rupture of Muscle and Tendon; and of Bruise."

We trust that the epitomè we have thus given of Mr. Miller's production will justify our commencing observation, that a reference to its pages entirely removed any feeling of its unnecessariness, induced by its title. We have much pleasure in recommending the work to all members of our profession, whether they be yet students of our art, or actively engaged in administering the inestimable benefits of medicine; whether devoted to an exclusive branch, or to general practice, much instruction and assistance will be gained from its perual, and frequent reference to its pages. The manner is unfortunately not always an exception from that which generally stains medical writings; a looseness and indistinctness of language often prevails, with occasionally a spirit of dictation which, however it may be excused in the lecture-room, is better avoided in a publication.

